# Effect of pristine graphene incorporation on charge storage mechanism of three-dimensional graphene oxide: superior energy and power density retention

**DOI:** 10.1038/srep31555

**Published:** 2016-08-17

**Authors:** Kiran Pal Singh, Dhrubajyoti Bhattacharjya, Fatemeh Razmjooei, Jong-Sung Yu

**Affiliations:** 1 Department of Energy Systems Engineering, DGIST, Daegu 42988, Republic of Korea

## Abstract

In the race of gaining higher energy density, carbon’s capacity to retain power density is generally lost due to defect incorporation and resistance increment in carbon electrode. Herein, a relationship between charge carrier density/charge movement and supercapacitance performance is established. For this purpose we have incorporated the most defect-free pristine graphene into defective/sacrificial graphene oxide. A unique co-solvent-based technique is applied to get a homogeneous suspension of single to bi-layer graphene and graphene oxide. This suspension is then transformed into a 3D composite structure of pristine graphene sheets (GSs) and defective N-doped reduced graphene oxide (N-RGO), which is the first stable and homogenous 3D composite between GS and RGO to the best of our knowledge. It is found that incorporation of pristine graphene can drastically decrease defect density and thus decrease relaxation time due to improved associations between electrons in GS and ions in electrolyte. Furthermore, N doping is implemented selectively only on RGO and such doping is shown to improve the charge carrier density of the composite, which eventually improves the energy density. After all, the novel 3D composite structure of N-RGO and GS greatly improves energy and power density even at high current density (20 A/g).

Energy and power densities are the main features, which distinguish the energy storage devices. In supercapacitors, also known as double layer capacitors, the movement of charge determines both the energy and power densities. Unlike batteries, supercapacitors store their energy in electrode-electrolyte interphase generated due to the application of voltage bias^1^,^2^,^3^,^4^. Another distinction of supercapacitors is their superior power density compared with batteries, which arises due to negligible resistance encountered during charge transfer. However, compared with batteries, supercapacitors possess much less energy density, and to improve this aspect, various studies have been performed, especially on carbon electrode, where it has been shown that improving the pore volumes (mico/meso) helps in enhancing the specific capacitance and energy density^5^,^6^,^7^,^8^,^9^. However, the destruction of carbon electronic structure due to pores incorporation, increases the resistivity in the carbon electrode, which ultimately causes a decrease in capacitance at higher current density, eventually dragging down the power density of the supercapacitor (P = V_i_^2^/4R, where P is maximum power output, R is resistance and V is applied voltage), and limiting its applications in many fields^1^,^10^,^11^,^12^. Eventually, in the race of gaining higher capacitance/energy density, carbon’s capacity to retain power density is generally lost due to defect incorporation and resistance increment in the carbon electrode. In this regard, we lack a common ground, where both the properties of supercapacitors can be exploited effectively.

Charge storage and utilization in supercapacitor devices, are highly dependent on the defect density of the carbon. It has been reported that the volumetric capacitance of the supercapacitors gets saturated, if surface area of carbon increases beyond a certain limit, especially when pores have thin walls similar to the thickness of graphene^12^. In recent studies, it was found that the interfacial capacitance gets limited by the quantum capacitance although the surface area of the graphene is increased. Therefore, experimentally it was observed that surface area-normalized capacitance of reduced graphene with surface area of 705 m^2^ g^−1^, is much higher than that of activated graphene oxide (GO) with surface area of 3100 m^2^ g^−1 ^13,14. Hence, it can be deduced that the improved surface area is not the only determining property, which induces higher capacitance into the graphene-like materials. Zhang *et al.*^15^ later showed that the superior volumetric capacitance in graphene-like materials is derived not only from the surface area, but also from the charge density. It was shown that capacitance at the point of zero charge (PZC) was uplifted after doping of nitrogen (N) into the graphene matrix, and this increase was credited to the superior charge density in N-doped graphene^15^. The five valence electrons in nitrogen’s outer shells impart n-type doping once N gets doped into the carbon framework. However, the extent of n-character or free electron density depends on the type of N doping in carbon, such as graphitic, pyridinic and pyrrolic. For instance, in graphitic nitrogen, three electrons among five valence electrons, participate in the formation of three σ bonds with three neighbor carbon atoms, one electron goes in the p_z_ orbital, and the fifth is an additional electron that actually occupies a π^*^-donor state, providing n-type doping to graphene. In contrast, pyridinic and pyrrolic nitrogen atoms are found near a carbon vacancy and so they have only two carbon atoms as neighbors. As a consequence, they contribute 2 electrons with only two σ bonds along with one electron in the pz orbital forming a π-bond with its neighbor, and thus leave two electrons as a lone pair. Imparting N-doping in carbon, can therefore have huge implication on its surface morphology as well as its electrochemical performance. Hence, it can be concluded that in carbon materials like graphene, one can achieve superior capacitance/energy density by simply increasing the charge carrier density as well as sheet exposure to electrolyte. Therefore, graphene can be an excellent candidate for obtaining higher energy density without significantly disrupting the power density.

Herein the major challenge is to incorporate N heteroatom into the graphene matrix as pristine graphene has a perfect structure, and it is difficult to introduce heteroatom without disrupting its lattice^16^. Nitrogen doping like other heteroatoms (oxygen, phosphorus, sulphur, and so on), distorts the carbon lattice, due to the difference in the electronegativity and bond length between C-C and C-N. These kinds of variances introduced by a dopant into the carbon lattice can distort the π electron cloud, which eventually hinders the in-plane electron movement on the graphene lattice. Furthermore, even though pristine graphene shows excellent promises in lowering the relaxation time and enhancing power density^12^,^17^,^18^, its applicability is limited due to restacking of graphene sheets, and pristine graphene has also comparatively less energy density than that of N-doped graphene^19^,^20^,^21^,^22^,^23^. Thus, it would be very effective if pristine graphene can be distributed into N-doped graphene oxide matrix, as a mono or a few layers of graphene sheets, for maximum power density. Therefore, in this work, a unique novel methodology has been developed for the first time to realize a new three-dimensional (3D) composite structure, where defect-free pristine graphene sheets (GSs) are homogeneously incorporated between defective N-doped reduced graphene oxide (N-RGO) layers as an efficient model for high performance supercapacitor. Here, GO serves as a sacrificial moiety in which heteroatom dopant and defects are introduced, which helps in improving the charge storage capability of the composite. On the other hand, dispersed GSs due to their superior conductivity and defect-free surface can drastically decrease the defect density and thus decrease relaxation time due to improved associations between electrons in GS and ions in electrolyte, greatly improving power density. Therefore, N-RGO-GS composite can be an excellent candidate for supercapacitor as it possesses properties, which can decrease relaxation time and increase capacitance while maintaining both high energy and power density. A similar concept of enhancing the overall capacitor performance has been reported before by using CNT and graphene oxide composite^24^,^25^,^26^,^27^. Recently Xu *et al.* showed a tremendous specific capacitance improvement after PANI incorporation in carbon aerogel. However the capacitance retention of the material was quite low and it retained only 66% of the initial capacity at 10 A/g^28^. However, due to the complexity of achieving defect-free and stable graphene sheets, incorporation of pristine graphene into graphene oxide has never been reported earlier.

Herein, we take the advantage of π electronic interaction of graphitic carbon to obtain a stable uniform 3D composite of N- RGO and pristine GS via freeze drying for generation of high surface area and macropores. Giving a 3D structure to the composite is essential to exploit the complete surface of GSs. To obtain this 3D structure by freeze drying, it is of greatest importance to identify the solvent, which can disperse and keep both graphene and GO sheets stable and be compatible for freeze drying process as well. Therefore, the use of normal organic solvents, which are generally being used for dispersing graphite, is out of question as they can introduce other functional groups onto graphene sheets during processing^29^ and are not compatible for GO dispersion and freeze drying as well. To deal with the situation, we have adopted a co-solvent (water-ethanol) system, based on Hansel solubility parameter (HSP)^30^, which can efficiently disperse both graphene materials and GO. In brief, firstly both graphite powder and GO were separately dispersed in water-ethanol mixture through sonication. With help of sonication, GSs were generated from graphite powder. After mixing both the solutions, the mixture was then freeze-dried to obtain 3D structure of graphene oxide-graphene sheet (GO-GS) composite. To carry out the N-doping and reduction of GO, the hydrazine vapor reduction of GO-GS was carried out to obtain N-doped reduced graphene oxide-graphene sheet (N-RGO-GS) composite as shown in Fig. 1 (For more details, see method section)^31^. This reduction process is preferred over a high temperature carbonization and other processes, due to the capability of this process to selectively incorporate N into GO matrix, without disrupting the pristine graphene matrix (Figure S1 of Supplementary Information (SI)). The supercapacitance performance of the N-RGO-GS composite shows a remarkable capacity retention even at current density as high as 20 A/g, as compared with N-RGO alone.

## Results and Discussion

**Figure 2:**
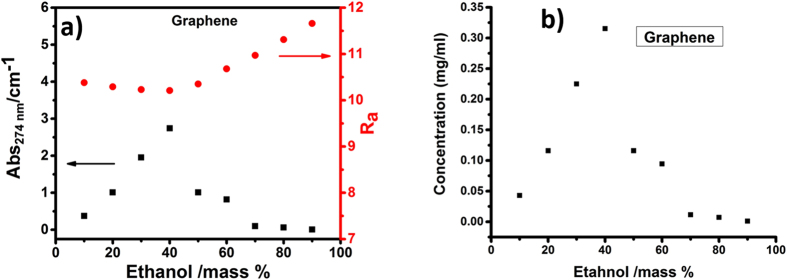
(**a**) Absorbance of the graphene in ethanol/water mixtures and their respective calculated Ra values according to Hansen solubility parameter and (**b**) graphene concentration variation with respect to ethanol concentration.

First, the efficacy of the water-ethanol co-solvent system for exfoliating graphite is evaluated. Figure 2a shows the absorbance determined from UV spectroscopy of the dispersed graphite in various ethanol-water compositions. It can be seen that the absorption spectra is highly dependent on the amount of ethanol used, and the best dispersion is obtained for the system consisting of 40% of ethanol. The R_a_ (HSP distance) curve shows the parabolic behavior with minimum at 40% ethanol concentration in water, which clearly illustrates that the adaptability of co-solvent system for graphite is maximum at this ethanol concentration. On the basis of cohesive energy, calculated by considering atomic and molecular bonding, such as dispersive δ_D_, polar δ_P_, and hydrogen bonding δ_H_, (see Table S1 of SI) of the solvent system, the solubility parameter required for the efficient dispersion of solute can be predicted. HSP for solvent and solute can be calculated by finding the level of their adaptation also known as HSP distance, R_a_,


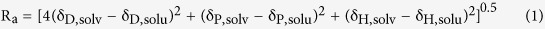



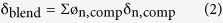


where R_a_ is the length of the vector from the point in Hansen space representing the graphene to the point representing the solvent (azeotrope). Therefore, the smaller the value of R_a_, the better the adaptation of solvent for solute. For the present system on the basis of R_a_ value, we have tried to determine the exact concentration of ethanol and water in the co-solvent system, required for the efficient dispersion of graphite. For our present system, 40% of ethanol in water was found to give the best dispersion of graphite. The concentration of graphite in 40% ethanol-60% water system is calculated to be 0.32 mg/ml as shown in Fig. 2b.

Quality of the dispersed graphene was further evaluated. Figure S2 of SI shows that dispersed sample mainly comprises of 1–2 layers of graphene. The electron diffraction pattern shows that the mixed solvent system does not disrupt the chemical integrity of the obtained graphene. From atomic resolution HR-TEM images, the lattice constant for graphene is found to be 0.25 nm, which is consistent with that of pristine graphene. On the other hand, Figure S3 of SI shows the dispersed GO morphology. It is seen that GO sheets are efficiently dispersed in water-ethanol azeotropic system like in pure water system.

**Figure 3:**
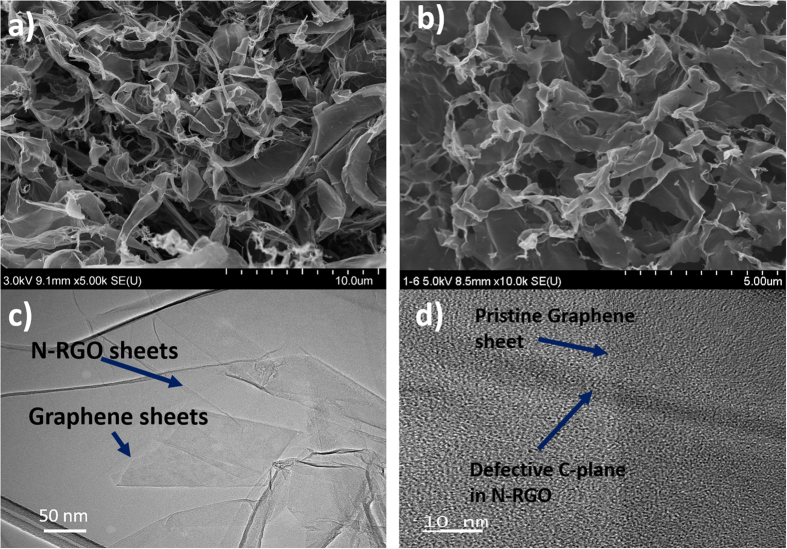
SEM images of (**a**) N-RGO-GS, (**b**) N-RGO, and (**c,d**) TEM images of N-RGO-GS.

Topographical features of the freeze-dried N-RGO-GS composite and N-RGO are shown in Fig. 3. SEM images for both the samples were quite similar, and a free standing 3D graphene structure can be seen clearly. This 3D structure can definitely provide maximum sheet exposure to the electrolyte ions due to the presence of macroscopic pores and is necessary to keep the sheets separated from each other. Interestingly, in TEM image for N-RGO-GS in Fig. 3c, it is found that N-RGO sheets are wrapped onto GS, which simply implies the existence of π-π interaction between two graphene materials. In solid phase, the restacking of graphene sheets occurs due to the presence of weak van der waals forces. However, once the graphite is stably dispersed in any solvent, this force has almost no effect between them as it is counterbalanced by the surface tension of the liquid. On the other hand, in the case of graphene oxide, due to incorporation of oxygen moieties they becomes hydrophilic in nature and can be easily dispersed in many aqueous solvents. Therefore, once graphene oxide is stably dispersed in azeotropic solvent (water:ethanol) as shown in the present study, upon addition of graphene sheets dispersed in the identical azeotropic solvent (water:ethanol), there may occur an interaction between graphene oxides and the graphene sheets due to lattice matching or π-π interaction associated with presence of graphitic lattice in both materials. Similar phenomena have been observed previously as well, where graphene oxide solution have been used as dispersant for the dispersion of the carbon nanotubes^32^,^33^. Hence, we can surmise that due to the presence of these weak forces, i.e. π-π interaction or lattice matching, the final product will be an composite of graphene oxides and graphene sheets. This morphology also illustrates the presence of GS between two RGO sheets. This kind of packing can keep the GSs separate and also offer efficient electrical transmission path in the composite. Figure 3d shows a HR-TEM image, where it can be seen that the prepared N-RGO-GS sample contains defective N-RGO sheets on the top of the pristine GS. Therefore, it can be predicted that the presence of stable pristine GSs can significantly enhance the electronic conduction of the composite materials. Herein, we would again like to mention that the graphene dispersion contains 1–2 layer of GSs and thus, the possibility of their restacking during addition of GO solution is very less as the dispersant used for GO is also the same concentration of ethanol-water solution, which can keep the overall solubility parameter of the solvent system constant. In addition, the π-π interaction between GSs and GO sheets can keep GSs stably dispersed in the solution^34^,^35^. For comparison we had also freeze-dried graphene dispersion only without graphene oxide, and it is found that the dried product consists of heavily stacked graphite sheets (Figure S4 of SI), which signifies the importance of incorporating graphene oxide for obtaining stable respective pristine graphene sheets.

**Figure 4:**
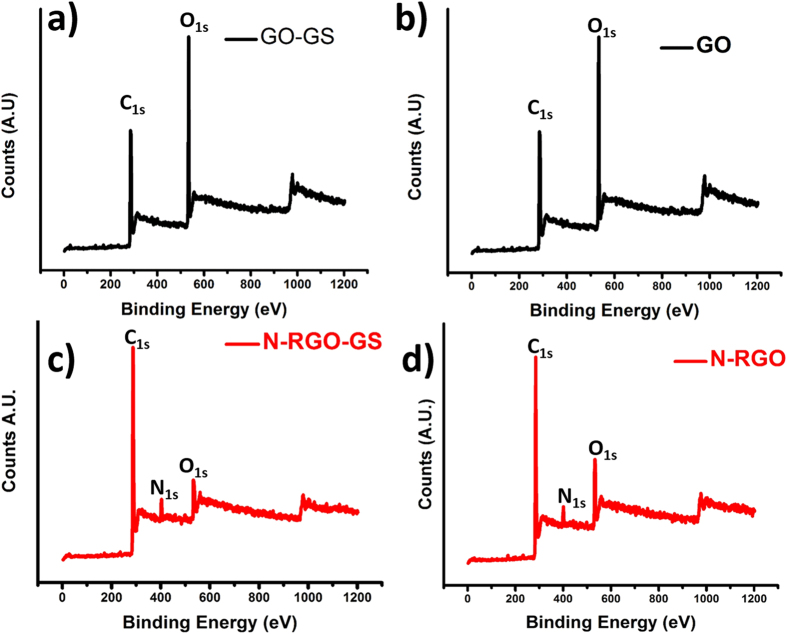
XPS survey scans of (**a**) GO-GS, (**b**) GO, (**c**) N-RGO-GS, and (**d**) N-RGO.

The effectiveness of present reduction process on the prepared composite material is analyzed by XPS analysis. Figure 4 shows the full XPS survey graphs for GO, GO-GS, N-RGO, and N-RGO-GS. High intensity oxygen signal in GO and GO-GS samples due to the presence of GO can be noticed. It can be seen that after hydrazine vapor treatment, C/O atomic ratio changes from 2.3 to 14.2, along with the introduction of 2.9% of nitrogen, indicating the simultaneous reduction and doping of GO in N-RGO-GS composite. As shown in Table S2 of SI, similar trend is observed in GO and N-RGO samples as well. Interestingly, lesser amount of carbon content is observed in GO and N-RGO (Table S2 of SI) as compared with GO-GS and N-RGO-GS, due to the presence of pristine GSs in the composites. High resolution of C1s spectra can be seen in Figure S5a of SI. Interestingly, intensity of C1, sp2 hybridized carbon, is found to increase after addition of graphene in N-RGO matrix, whereas C2, sp3 hybridized carbon, decreases due to pristine GS in N-RGO-GS. On a closer look after N1s deconvolution (Figure S5b of SI) it is found that in both N-RGO and N-RGO-GS, N heteroatom mainly got doped as a pyrrolic or amine species (ca. 399.5 eV)^36^. In addition, the overall peak intensity of nitrogen content is found to be decrease and this decrement can be attributed to the presence of N-free pristine GSs in N-RGO-GS composite. Even though there are various factors which influence the performance of the supercapacitors, N-doping is a key factor, which improves binding energy of the ions in the electrolyte with the electrode. The binding energy enhancement would contribute to the capacitance increase because larger binding energy allows a larger number of ions to be accommodated on the electrode surface^37^. If we consider the electronic charge distribution around various types of N-dopant, pyrrolic and pyridinic nitrogen can impart high charge concentration in the carbon lattice due to the availability of 2 lone pair of electrons in their outer orbital, as compared to graphitic nitrogen^38^. In addition, pyrrolic-N is known to contribute greatly to electrochemical behavior as it reduces the band gap of carbon. This means that carbon framework with a pyrrol nitrogen-containing group at the edge of graphene layer has high charge mobility in a carbon matrix and excellent donor-acceptor properties^39^. Hence, we can surmise that the presence of pyrrolic nitrogen in the carbon lattice can improve the overall charge storage capacity of the carbon electrode.

To study the crystal structure, Raman analysis was performed on the prepared samples (Figure S6 of SI). Interestingly, very pronounced G band and 2D band are observed in N-RGO-GS in comparison to N-RGO, which clearly indicates the efficient incorporation of GSs into GO matrix. BET surface area of the prepared samples was also evaluated as shown in Figure S7 of SI. An obvious decrement in mesopores volume and surface area, from 0.79 to 0.51 cm^3^g^−1^ and from 190 to 171 m^2^g^−1^, is observed after graphene sheet incorporation. However, micropore volume is found to increase in N-RGO-GS (Table S3 of SI). This variation in surface area and pore volume can be attributed to the insertion of GSs between two graphene oxide layers as shown in Fig. 3.

**Figure 5:**
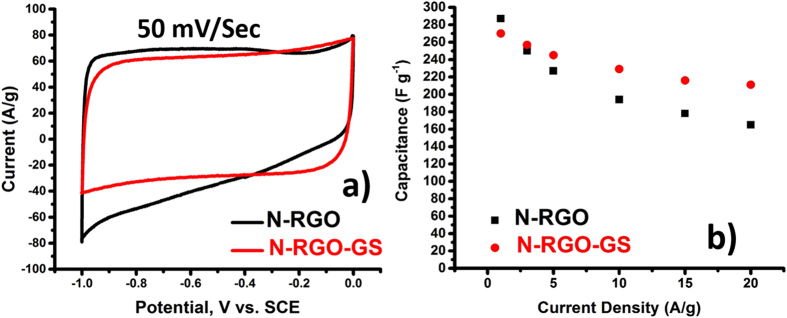
(**a**) CV scans at 50 mV/sec and (**b**) relative capacitance retention as a function of current density for N-RGO and N-RGO-GS electrodes.

The electrochemical testing was performed in a three-electrode setup by keeping Pt as a counter and SCE as a reference electrode. The effect of GS addition on the electrochemical behavior can be easily seen from the CV performance in Fig. 5a. A nearly perfect rectangular shape is obtained for N-RGO-GS sample, whereas a current suppression, indicating a slow discharge current release during voltage reversal, is observed for N-RGO sample at 50 mV/sec scanning. The gravimetric capacitances of the samples measured from charge-discharge curve are shown in Figure S8 of SI. To our surprise, it is found that although N-RGO-GS possesses less gravimetric capacitance than N-RGO at 1 A/g, its capacitance gets much better than N-RGO as the current density increases as seen in Fig. 5b. This indicates that to achieve desirable supercapacitance performance, it is of utmost importance to improve surface area without losing the conductivity of the carbon, which can decrease relaxation time. For comparison, the capacitance on freeze-dried graphite particles was also measured (Figure S9 of SI). These particles are found to possess much less capacitance due to very low defect density although their capacity retention is very high even at current density of 20 A/g (Figure S9b of SI). This also shows that a 3D uniform composite structure of both highly conductive GSs and high surface area N-RGO with high charge carrier density is necessary as an efficient model for high performance supercapacitor.

**Figure 6:**
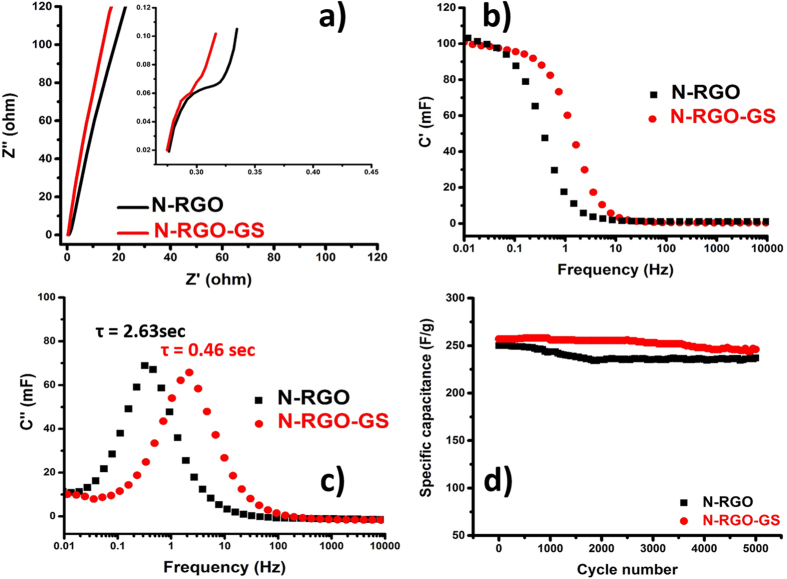
(**a**) Nyquiste plots, (**b**) C′ and (**c**) C″ vs. frequency plots, and (**d**) cyclic stability at 3 A/g of N-RGO and N-RGO-GS.

To understand this excellent stability in capacitance in N-RGO-GS as compared with N-RGO at higher current density, we have carried out the impedance analysis on the samples. Figure 6a shows the Nyquist plots of both the samples. Undoubtedly N-RGO-GS shows much better charge transfer conductance than N-RGO at higher frequency. A near ideal vertical graph without any high frequency RC (resistor-capacitor) loop is observed for N-RGO-GS unlike N-RGO. This indicates very good electrode contact throughout the N-RGO-GS sample facilitated by the presence of pristine graphene in it^40^. To get the better perspective of the capacitance behavior, the complex capacitance plots were plotted from impedance spectra (Fig. 6b,c). It can be seen from C’ vs frequency plot that both electrodes reach their maximum capacitance at lower frequency. However, the N-RGO-GS maintains its capacitance up to a much higher frequency (0.26 Hz) as compared with N-RGO electrode (0.07 Hz). The irreversible energy dissipation and relaxation process are judged from imaginary part capacitance (C”). The relaxation time constant ‘τ’ is calculated from the f_max_ of Fig. 6c. It can be seen that N-RGO-GS shows τ = 0.46 sec, whereas for N-RGO it was found to be 2.63 sec. Hence, after addition of GSs, the ion transport and rate constant get much better, and this in turn results in better power output for N-RGO-GS^41^.

As a result, from the lower relaxation time and higher frequency response of N-RGO-GS as compared with N-RGO, it can be understood that at lower frequency alternating current (AC) field, almost all the polarized ions in N-RGO are fully aligned, and hence N-RGO possesses better capacitance than N-RGO-GS. It is necessary to mention here that the defect density in N-RGO is much higher (see Figure S6 of SI) as compared to N-RGO-GS, which enables much higher charge storage in this material at lower frequency/current density. However, as the AC frequency increases, most of the polarized ions in N-RGO cannot follow the higher frequency field at each direction reversal, due to poor conductivity of this sample as compared with its counterpart. As the high-frequency AC field changes direction faster, these ions “relax” to nonaligned positions, where N-RGO cannot store energy as much as N-RGO-GS can. Hence, a decrement in the N-RGO performance is observed at higher frequency^42^. Impact of GS incorporation on the energy and power density retention of N-RGO-GS can be clearly seen from the Ragone plot in Figure S10 of SI. The cyclic stability of both the samples is also tested upto 5000 cycles (Fig. 6d), and only a mild capacitance fading is observed, signifying that the pore system of the prepared system can remain intact for a longer cycling period. For the sake of comparison, a comparative table portraying the capacitance performance of other graphene oxide composites and current N-RGO-GS is shown in Table S4 of SI. It can be seen that all the prepared materials shown in earlier works possess a very high surface area as compared with our sample prepared in the present manuscript, but the capacitance performance of our N-RGO-GS is equally comparative to others. Furthermore, we have also compared the capacity retention for various graphene and carbon composites and N-RGO-GS composite, in Table S4 of SI. It can be seen that the capacity retention for the N-RGO-GS material (~88%) is far better than that of the previously reported materials. This indicates the importance of introducing a defect free graphene lattice into the N-RGO framework. This clearly proves the point that the surface area is not the only determining factor for enhancing the capacitance performance. Hence, if the material is designed with high charge carrier concentration and the surface area of the graphene sheets can be fully exploited, an excellent capacitance with superb energy density without sacrificing power density can be achieved.

**Figure 7:**
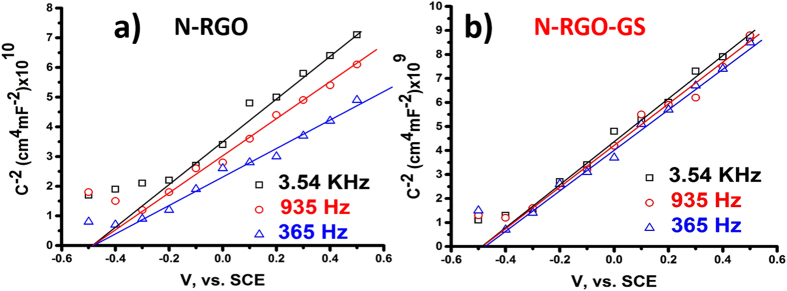
Mott-Schottky plots for (**a**) N-RGO and (**b**) N-RGO-GS.

To get a better picture of the origin of this phenomenon, Mott-Schottky plots are obtained for N-RGO and N-RGO-GS (Fig. 7). Before carrying out the impedance analysis, CV curves for both the samples were obtained in the potential range of −0.5 to 0.5 vs. SCE in 2.0 M KCl. It can be seen no faradic process occurs in the used potential range and hence the obtained capacitance can only attributed to the EDLC (Figure S11 of SI). A frequency dependent Mott-Schottky plots obtained for N-RGO and N-RGO-GS signify the spreading of localized states in the whole range of energies, which causes a slow response of charge carriers in the depletion region to the applied alternating potential^43^,^44^,^45^,^46^,^47^. As N-RGO possesses much higher amount of defects and oxygen content, it is reasonable to conclude that the carbon surface is more resistive and therefore possesses such frequency dependence behavior. Therefore, it can be established that due to the presence of pristine graphene, the presence of defects has been reduced to a greater extent, which has increased the AC response of the charge particles. This, in return gives rise to lower relaxation time and hence increasing the power and energy output of the N-RGO-GS samples at higher frequency.

In conclusions, herein, with the motive of finding a common ground of improving energy density without significantly losing the power density and proving the importance of defect-free conductive surface in supercapacitors, we have developed for the first time a novel uniform 3D high surface area structure of N-doped reduced graphene oxide–pristine graphene sheet (N-RGO-GS) composite, via simple freeze drying process, using a common ethanol-water azeotrope solvent. In the resultant novel 3D composite, the graphene sheets are present between two graphene oxide layers, and this kind of packing can keep the graphene sheets separate, thus offering efficient electrical transmission path in the composite scaffold with high surface area. The well-separated sheet structure provides much higher surface accessibility to the electrolyte ions, improving the electrolyte-electrode interaction. Furthermore, it was found that N-doping induces n-type charge carriers and helps in increasing the capacitance (energy density) of the N-RGO-GS. On the other hand, due to the incorporation of highly conductive pristine graphene, the defect density, which affects the frequency response of the capacitance, has been reduced, causing a drastic reduction in relaxation time. Therefore, much improved overall supercapacitance performance is observed even at higher current density (20 A/g), illustrating superior energy density retention. Through this study, we have also shown that in addition to higher surface area, a defect-free conductive surface is very essential for obtaining an excellent candidate for supercapacitor. This facile method of incorporating pristine graphene into disordered graphene oxide is expected to have huge implication towards the development of high efficient supercapacitor and battery materials in the energy storage field and can definitely open new dimensions in both synthetic and electro-chemistry of new carbon composite materials.

## Methods

### Material synthesis

#### Synthesis of Graphene Oxide

The graphene oxide was synthesized using improved Hummers method. In brief 3.0 gm of graphite (Alfa Aesar, 325 mesh), was mixed with 18.0 gm of KMNO_4_ in H_2_SO_4_:H_3_PO_4_ (9:1) solution. The obtained mixture was then stirred for 12 h at 50 °C. After getting brownish colour, 400 ml ice was added to the solution followed by dropwise addition of 3 ml H_2_O_2_. Obtained yellowish solution was then stirred for 2 h more. The final product was then obtained by centrifuging and washing with HCl and water. The obtained graphene oxide (GO) product was then dried at 70 °C overnight.

#### Preparation of pristine graphene-graphene oxide composite

Firstly, 10 mg graphite powder was dispersed in 50 ml ethanol/water mixture with 40 vol% ethanol volume, using a bath sonicator operated at low power for 8 h continuously. The obtained dispersion was then centrifuged at 3000 rpm for 30 mins, and the supernatant was collected. On the other hand, the GO product was dispersed in the same azeotropic solvent (50 ml ethanol/water mixture with 40 vol% ethanol volume), by 2 h of sonication for 25 mg GO. The GO dispersant was then centrifuged at 4000 rpm for 45 mins to get stable GO solution. To obtain the final composite, both graphene and graphene oxide solutions are then mixed together under sonication for 30 mins. The obtained composite solution was then freeze-dried in a freezer (operated at −40 °C) and dryer (operated at 5 m torr) (Ilshin Bio Ltd, Korea). The obtained graphene oxide-graphene sheet product is termed at GO-GS composite. For comparison, only GO dispersion was also freeze-dried by keeping all the conditions similar to those of GO-GS. In addition, we have tried to synthesize only 3D graphene by keeping all the conditions same as above, but in absence of graphene oxide.

#### N-doping and reduction of GO

The reduction of GO was carried by vapor hydrazine reduction. In brief, the GO or GO-GS sample, kept in a separate beaker, was treated with hydrazine vapor generated by the blowing Ar gas into 10 ml 50% hydrazine hydrate (Sigma Aldrich) for 8 h. During reduction process the samples were heated at 60 °C to stop the condensation of hydrazine on carbon samples. The completion of reduction was judged by the change of brown color of GO to black color of RGO. The reduced hydrazine-treated samples were then termed as N-RGO and N-RGO-GS for the corresponding GO and GO-GS, respectively. At the same time, N-doping was realized on the reduced GO during hydrazine process.

### Material characterization

The morphology and microstructure of the obtained samples were investigated by scanning electron microscopy (SEM) Hitachi (S-4700, Hitachi, Japan) microscope, Transmission electron microscopy (TEM) EM912 Omega and High resolution-TEM (HR-TEM) JEOL FE-2010 microscope. X-ray photoelectron spectroscopy (XPS) analyses were carried out with an ESCALAB 250 XPS System using a monochromated Al Kα (150 W) source. Raman spectra were recorded with a Renishaw spectrometer using an Ar ion laser (λ = 514.5 nm). The absorption spectra of the samples were recorded by using an Ultraviolet-Visible-Near Infrared Spectrophotometer (CARY 5000) manufactured by Agilent Technology. The specific surface areas of all the samples were measured with ASAP 2020 Physisorption Analyzer (Micrometrics, USA) at −196 °C, based on the Brunauer– Emmett–Teller (BET) method from nitrogen adsorption data in the relative pressure range from 0.05 to 0.2. Total pore volumes were determined from the amount of gas adsorbed at the relative pressure of 0.99. The pore size distribution was derived from adsorption branches by the Horvath-kawazoe (HK) report.

### Electrochemical Characterization

All the electrochemical measurements were carried out with a Biologic electrochemical workstation (Biologic VSP). N-RGO and N-RGO-GS carbon electrodes were fabricated as follows. The electrode slurry was prepared by mixing 90 wt% active materials and 10 wt% polyvinylidene fluoride (PVdF) binder in a mortar and pestle using a few drops of N-methylpyrrolidinone (NMP) as a solvent. The resulting uniform slurry was then coated onto a nickel foam current collector (1 cm^2^) and dried at room temperature overnight, followed by further drying at 80 °C in a vacuum oven for 12 h. The sample loading in all samples are kept constant at 10 mg. The electrochemical performances of the electrodes were tested using a three-electrode configuration in aqueous 6.0 M KOH electrolyte, where prepared carbon sample acts as a working electrode with SCE as a reference and Pt wire as a counter electrode. Cyclic voltammetry (CV), galvanostatic charge–discharge (CD) and electrochemical impedance spectroscopy (EIS) methods were employed for measurement of the electrochemical performance. The CV and CD curves were recorded between −1.0 to 0.0 V at potential scan rates of 10, 50 and 100 mVs^−1^ and at current densities of 0.1 to 1.0 Ag^−1^. The EIS measurements were carried out in the frequency range from 10 KHz to 100 mHz with a sinusoidal amplitude of 10 mV. The alternating current (AC) complex impedance was measured based on the following equation Z* = Z′−iZ″. The complex AC capacitance C* = C′−iC″ was calculated from the impedance data, as C′ = Z″/ω|Z|^2^ and C″ = Z′/ω|Z|^2^, where ω = 2πf, and f is frequency. The relaxation times τ = 1/fm were calculated from the relaxation frequencies fm, corresponding to the C″ maxima.

The energy density and power density of the electrodes at various current density is calculated using the following equations:


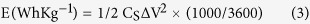



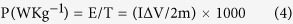


where E is the specific energy density, Cs is the specific capacitance, ΔV is the potential range, P is specific power density and T is the time of discharge. For the measurement of the interfacial capacitance, a three-electrode configuration was assembled using Pt plate of surface area 5 cm^2^ as a counter electrode, SCE as a reference electrode and N-RGO or N-RGO-GS sample as a working electrode. In order to make the working electrode, carbon paste, consisting of 95% active material, 5% PvDF and NMP, was painted over the Ti foil, whose edges are already protected with the scotch tape. The uncoated part of the electrode was isolated with Teflon coating. For ensuring low ohmic resistance, the leads were attached to the working electrode using silver paints. The electrolyte was a 2.0 M KCl solution without any additive and was purged with N_2_ gas for 1 h before the measurement. All the electrodes were partially immersed into the electrolyte, and the capacitance was measured using electrode impedance spectroscopy. The electrode impedance spectroscopy was performed by using a sinusoidal signal with amplitude of 10 mV over a frequency range of 100 kHz–10 Hz and a scanning potential range of −0.5 to 0.5 V versus a SCE reference electrode at a step size of 0.1 V with a 600 s equilibration time allowed at each step.

## Additional Information

**How to cite this article**: Singh, K. P. *et al.* Effect of pristine graphene incorporation on charge storage mechanism of three-dimensional graphene oxide: superior energy and power density retention. *Sci. Rep.* **6**, 31555; doi: 10.1038/srep31555 (2016).

## Supplementary Material

Supplementary Information

## Figures and Tables

**Figure 1:**
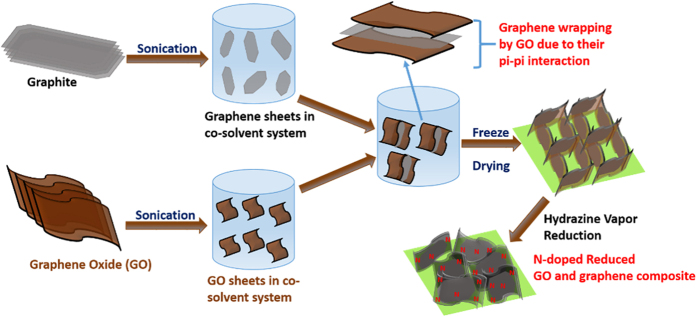
Schematic representation of novel 3D N-RGO-GS composite.
