# Supplementary material for: Effect of pristine graphene incorporation on charge storage mechanism of three-dimensional graphene oxide: superior energy and power density retention
Source: Sci Rep. 2016 Aug 17;6:31555. doi: 10.1038/srep31555 (PMC4987628; doi:10.1038/srep31555)
Supplement: Supplementary Information [file srep31555-s1.pdf]

Supplementary Information

Effect of pristine graphene incorporation on charge storage mechanism of three-dimensional graphene oxide: superior energy and power density retention

Kiran Pal Singh, Dhrubajyoti Bhattacharjya, Fatemeh Razmjooei & Jong-Sung Yu*

Department of Energy Systems Engineering, DGIST, Daegu 42988, Republic of Korea

*To whom correspondence should be addressed. E-mail: jsyu@dgist.ac.kr

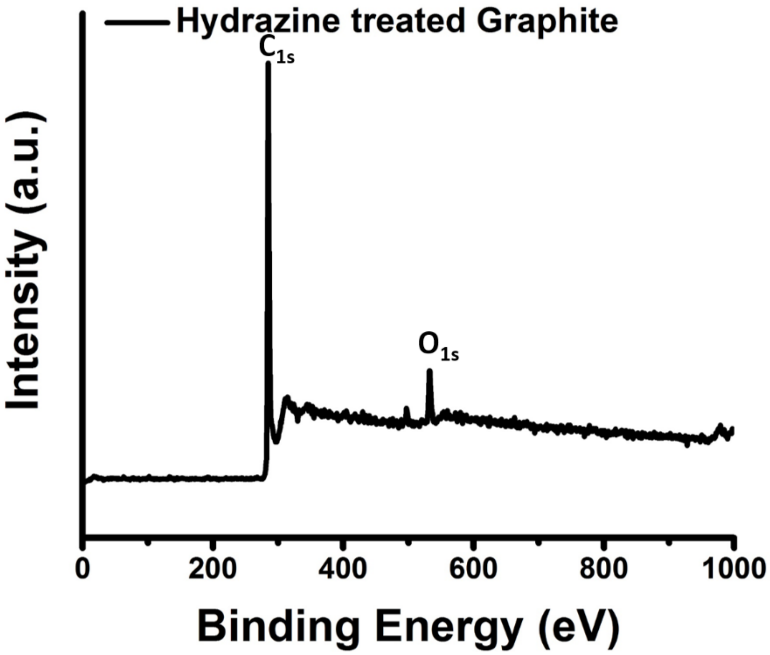

Figure S1. XPS of hydrazine-treated freeze-dried pristine graphite powder.

For comparison we have carried out freeze-drying on the ethanol-water exfoliated graphite powder. The obtained powder was then treated with hydrazine vapors to see the effect of hydrazine reducing atmosphere on the chemical properties of the pristine graphite. It is found that hydrazine vapor treatment have almost no effect on the graphite powder. Hence we can surmise that hydrazine selectively reacts with the graphene oxide only in GO-GS composite and N-doping occurs selectively on GO, leaving graphene structure unaffected.

Table S1. HSP values for ethanol-water solvent and graphene

	δ_D $(MPa^{0.5})$	δ_P $(MPa^{0.5})$	δ_H $(MPa^{0.5})$
Ethanol[1]	18.1	17.1	16.9
Water[1]	15.5	8.8	19.4
Graphene[2]	18.0	9.3	7.7

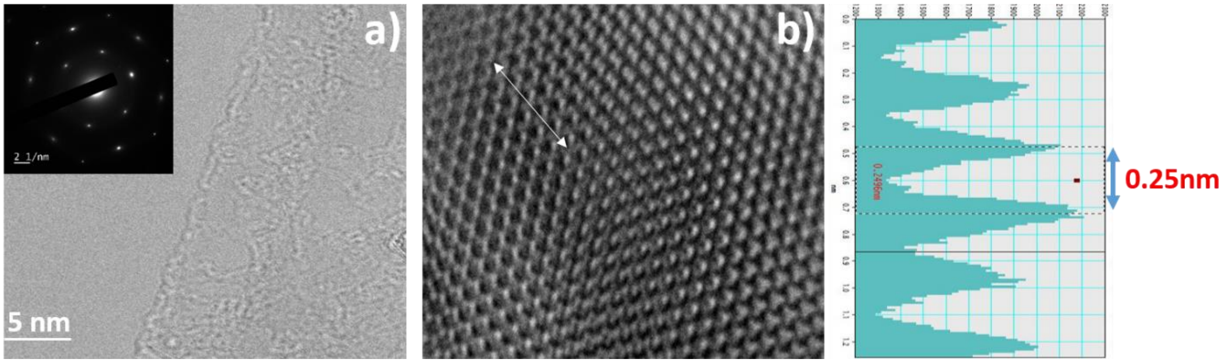

Figure S2. a) HR-TEM image (inset: electron diffraction pattern) and b) atomic resolution HR-TEM image of ethanol/water co-solvent exfoliated graphene along with corresponding line profile.

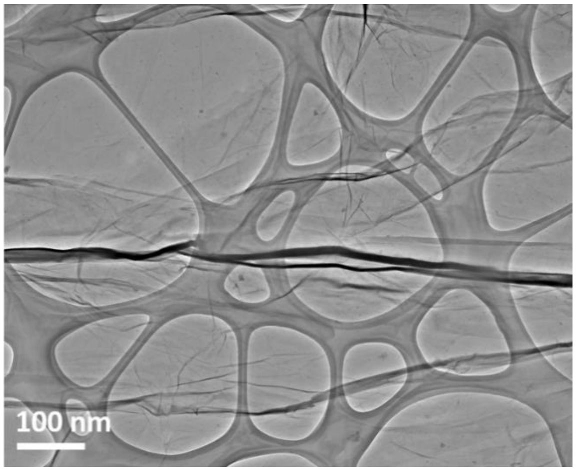

Figure S3. HR-TEM image of graphene oxide dispersed in water-ethanol azeotrope.

Table S2. Atomic composition of N-RGO and N-RGO-GS

Sample	C1s	O1s	N1s
**GO**	64.8	35.2	0
**N-RGO**	85.5	11.3	3.2
**GO-GS**	69.1	30.9	0
**N-RGO-GS**	90.7	6.4	2.9

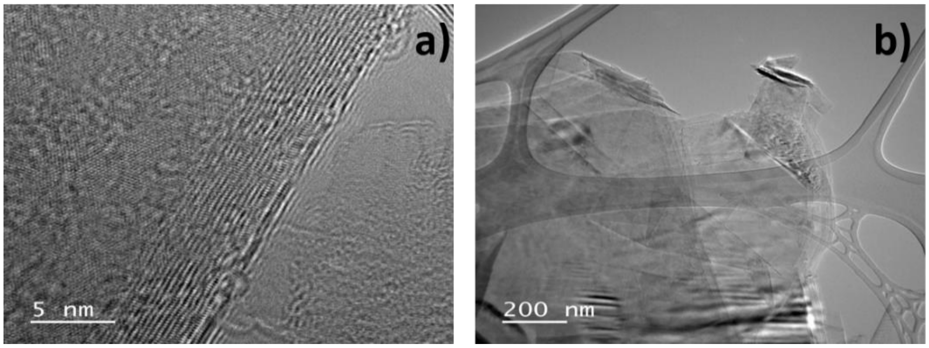

Figure S4. a) HR-TEM and b) TEM images of graphite particles obtained after sonication and freeze drying.

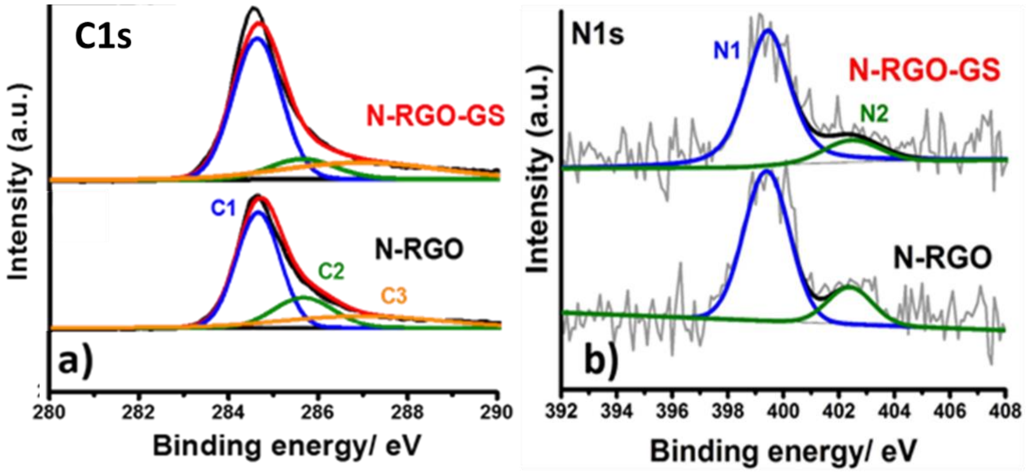

Figure S5. Deconvoluted spectra of a) C1s and b) N1s for N-RGO and N-RGO-GS

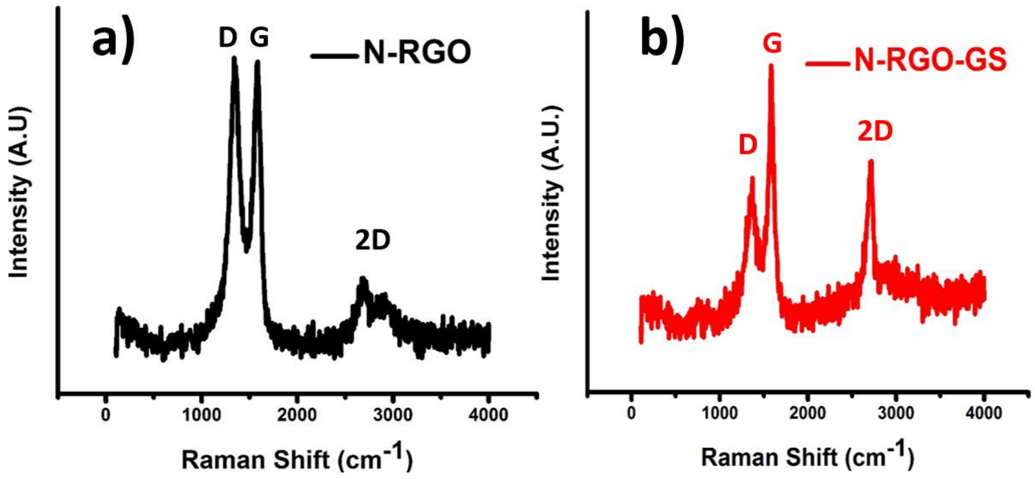

Figure S6. Raman spectra of a) N-RGO and b) N-RGO-GS.

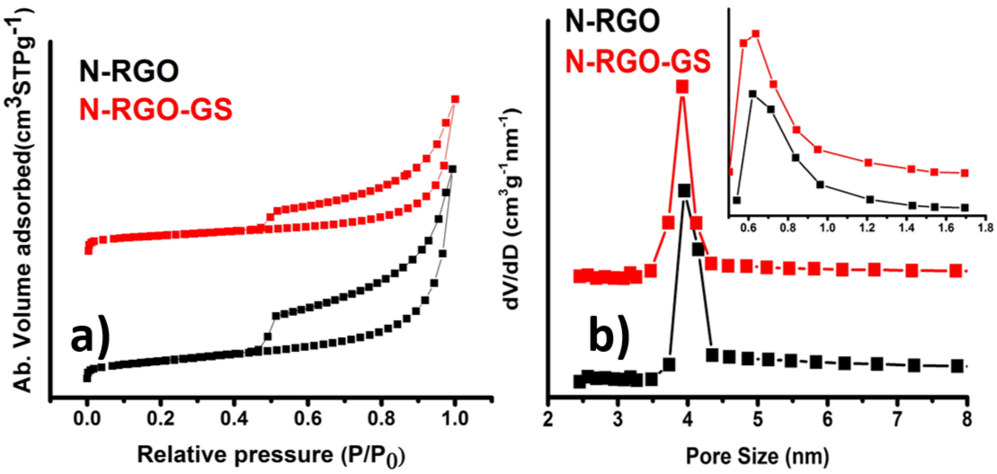

Figure S7. a) Nitrogen adsorption-desorption isotherms and (b) the corresponding pore size distribution curves for N-RGO and N-RGO-GS.

Table S3. Structural characteristics by nitrogen sorption data of N-RGO and N-RGO-GS

Sample	BET total surface area (m^2g^{-1})	Mesopore surface area (m^2g^{-1})	Pore volume (cm^3g^{-1})	Mesopore volume (cm^3g^{-1})	BJH pore size (nm)
**N-RGO**	190.38	162.19	0.88	0.79	4.01
**N-RGO-GS**	171.56	125.35	0.68	0.51	3.95

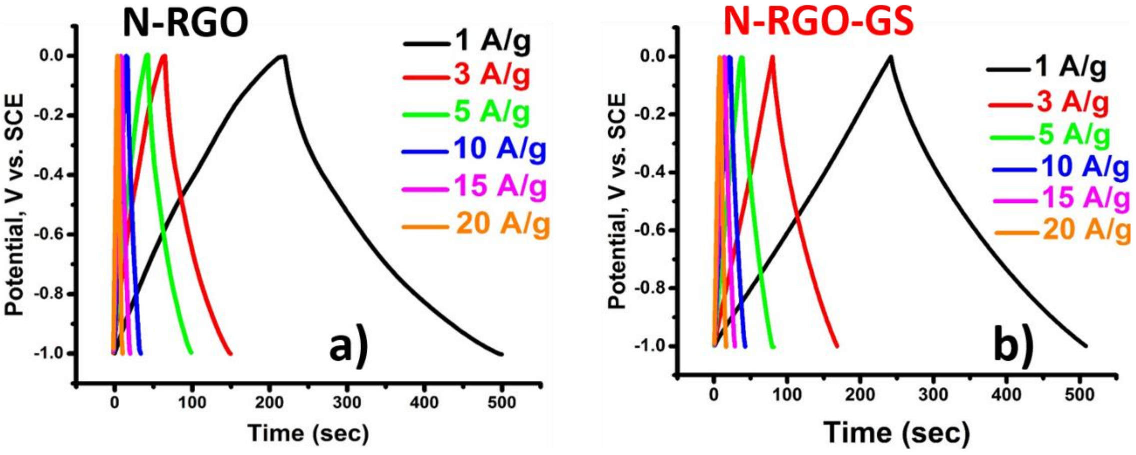

Figure S8. Charge-discharge profiles of a) N-RGO and b) N-RGO-GS at different current densities.

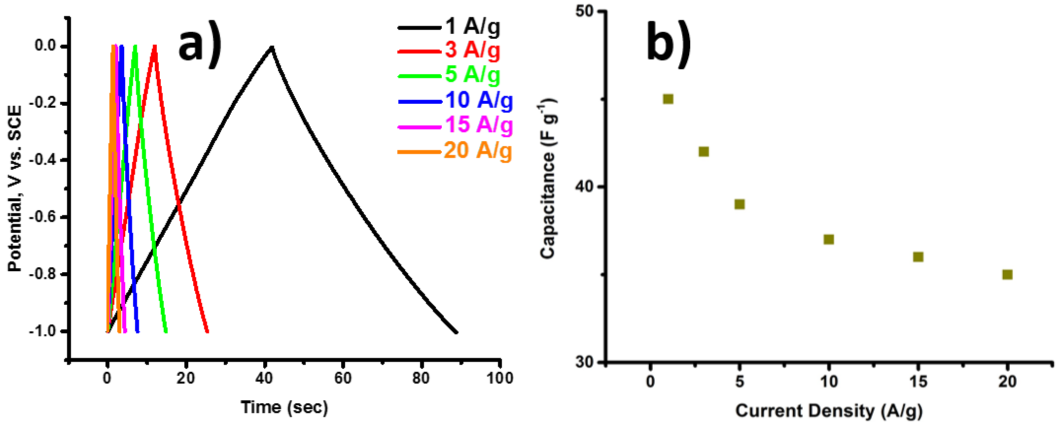

Figure S9. a) CD and b) relative capacitance retention as a function of current density for freeze-dried graphene.

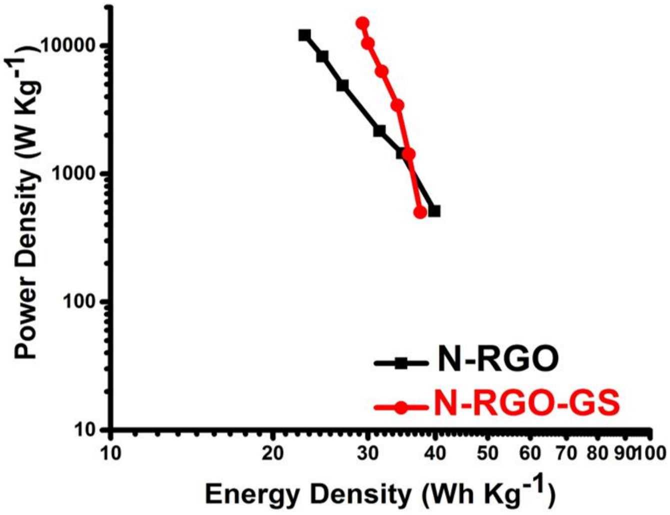

Figure S10. Ragone plots of N-RGO and N-RGO-GS.

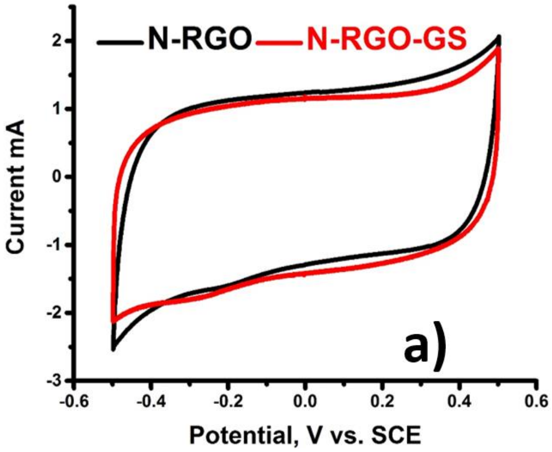

Figure S11. CV curves for N-RGO and N-RGO-GS at 10 mV/s measured in 2.0 M KCl solution.

Note:

Mott-Schottky (M-S) plots have been shown for the semiconductor/electrolyte phases to determine various physical and chemical properties of the semiconductor[3,4]. Here, we have use the plots to understand the effect of frequency on the space charge capacitance, C_{sc} at various voltage bias. For the modeling of C_{sc}, it has been assumed that it is in series with the Helmholtz capacitance (C_H) and diffusion capacitance (C_{Diff}). Therefore, the total EDL capacitance can be written as:

$$1/C_{EDL} = 1/C_{SC} + 1/C_H + 1/C_{diff} \qquad \text{---------} \; 1$$

As the concentration of electrolyte is very high, the value of 1/ C_{Diff} can be neglected. Therefore, for M-S analysis it is assumed that the total capacitance is mainly derived from the C_{sc}, and its value should be less than C_H. From the above M-S plots, the flat band potential (V_{FB}) (supposed to define the potential of zero charge) and the carrier density can be easily calculated using the following relations, due to the presence of n-type dopant. The M-S relation will be as follows.

$$1/C^2_{SC} = ((2/e\varepsilon\varepsilon_0 N_D)) \text{ X } (V - V_{FB} - \kappa T/e) \qquad \text{---------} \; 2$$

where C_{sc} is the capacitance of the space-charge region, ε is the dielectric constant of the semiconductor, ε_0 is the permittivity of free space, e is the electron charge (1.602 X 10^{-19} C), N_D is donor carrier densities, V is the applied potential, κ is the Boltzmann constant (1.38 X 10^{-23} J K^{-1}), and T is the absolute temperature in which κT/e can be negligible because it is only ~25 mV at room temperature. V_{FB} can be determined from V_O, the point of intersection of a C^{-2} vs V plot with the V-axis:

$$V_0 = V_{FB} + \kappa T/e \qquad \text{---------} \; 3$$

As can be seen in Figure S7, it is possible to get the knowledge about V_{FB} for both the studied samples, as at all frequencies, C^{-2} vs V curves are intersecting at one point. However, the information regarding N_D has been lost in N-RGO, due to the variation of C^{-2} vs V slope with the frequency[5]. There have been various studies, which have included multiple factors for describing the frequency dispersion of capacitance[5-8]. Since in the present case, electrode contacts were made up of silver paint and the electrodes are made up of N-RGO and pristine graphene sheets, the dispersion due to ohmic resistance or the bulk resistance can be ruled out. Secondly, the effect of non-uniform distribution of a.c. near the electrode surface, such as the edge effects, seepage of solution between the electrode and its insulation, or due to the roughness of the electrode surface, on the frequency dependence has to be ruled out. Here, edge effect is negligible due to the covering of electrode edges with the scotch tape. The seepage between teflon coating and electrolyte was not observed[9]. To guarantee the negligible effect of roughness factor, the pastes were spread on the electrode surface using a doctor blade followed by roll pressing. Another important factor that can affect the frequency dispersion in M-S plot is the formation of Helmholtz layer at the semiconductor/electrolyte interface. In this case the equation 2 is no longer valid and extra layer capacitance (C_L) has to be added into the above equation if it is voltage independent.

$$1/C^2_{SC} = 1/C^2_L + ((2/e\varepsilon\varepsilon_0 N_D)) \text{ X } (V - V_{FB} - \kappa T/e) \qquad \text{---------} \; 4$$

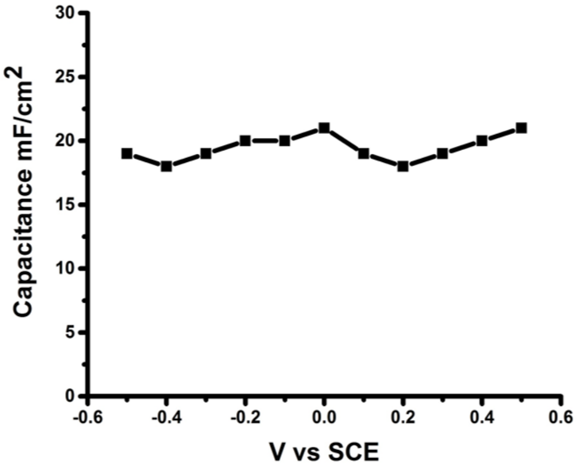

Figure S12. Interfacial capacitance for Pt foil.

To understand the interfacial capacitance behavior on the electrolyte side we have measured the interfacial capacitance using Pt as working electrode. The average capacitance was found to be around 19 µF, and it can be seen that the capacitance is voltage independent[10]. It can be seen that the C_H is very high as compared to the C_{sc} (Figure 6), and hence the contribution of C_L can be neglected in the frequency dissipation of the material.

There have been several reports pointing out the frequency dependency of the dielectric constant, ε, in the space charge layer due to the emergence of dipolar relaxation at various frequencies and voltages. In these cases, a linear C^{-2} vs V plots with a slope depending on frequency and converging to one common point on the V-axis is usually obtained, similar to the one obtained in present N-RGO case[5,9,11]. It has been found that this kind of frequency dependence of capacitance of semiconductor electrode originates not only from the layer adjacent to the electrolyte, but also from the number of layers spread throughout the bulk of the semiconductor[11]. As N-RGO possesses much higher amount of defects as well as oxygen content, it is reasonable to conclude that the carbon surface is more resistive and therefore possesses such kind of dependence behavior.

Note 1. Justification for need of pristine graphene layers and N dopant in supercapacitor

Supercapacitors are known for possessing high power density. However, their efficacy to store power is generally compromised in the process of improving their energy density. The destruction of carbon electronic structure, due to pores or defects incorporation, increases the resistivity in the carbon electrode, which ultimately causes a decrease in capacitance at higher current density, eventually dragging down the power density of the supercapacitor ($P = V_i^2/4R$, where P is maximum power output, R is resistance and V is applied voltage). Therefore, it is highly desirable to maintain the electronic conductivity of the material in addition to improving its surface area. As is well known that single graphene sheets are the most conductive form of carbon, in this work we have tried to keep the pristine graphene sheets stably dispersed into the N-doped reduced graphene oxide matrix to improve the conductivity and reduce the defect density of the composite N-RGO-GS. The effect of pristine graphene sheets on the performance of the supercapacitor can be judged from the lower relaxation time and higher frequency response after defect-free graphene sheet incorporation in N-RGO-GS. The defect density in N-RGO is much higher (see Figure S6 of SI) as compared with N-RGO-GS, and thus, the N-RGO enables higher charge storage in this material at lower frequency/current density. However, as the AC frequency increases, most of the polarized ions in the N-RGO cannot follow the higher frequency field at each direction reversal due to poor conductivity of this sample as compared with its counterpart (N-RGO-GS). As the high-frequency AC field changes direction faster, these ions "relax" to nonaligned positions, where N-RGO cannot store energy as much as N-RGO-GS can. Hence, a decrement in the N-RGO performance is observed at higher frequency and higher current density which results in a poor power density of the material. Impact of GS incorporation on the energy and power density retention of N-RGO-GS can also be clearly seen from the Ragone plot in Fig. S10 of SI.

The interplay or the exact role of N-doped graphene oxide can be understood from the following basic supercapacitor expressions.

$$C = Q/V \qquad \text{--------- 5}$$

and, $$E = \tfrac{1}{2}\, CV^2 \qquad \text{--------- 6}$$

Hence, $$E = \tfrac{1}{2}\, QV \qquad \text{--------- 7}$$

where Q is the charge stored on the capacitor plates, V is the applied bias potential, C is the capacitance and E is the energy density. Here, it can be seen that E and Q are proportional to each other. As N-doping is known to improve the charge carrier density of the material, it can be surmised that it will also help in improving the energy density of the material. Similar phenomena have already been observed by various other groups as well.[12-14] Furthermore, it has been known that the manipulated local electronic structures of carbon due to N-doping, allows for enhanced binding with ions in the solution, and this exceptional feature has been utilized for high-capacity energy storage devices. Hence, in present manuscript the main motive of incorporating N heteroatom into the graphene matrix was to improve its charge carrier density, which in turn will improve the capacitance value. This in turn does not require the need for incorporation of pores into the graphene matrix.

In usual supercapacitor devices, N-doping helps in improving the energy density of the material by improving the charge carrier concentration. However, it also imparts distortion into the carbon lattice, which in turn decreases the charge mobility into the carbon matrix. Therefore, even though N-doping shows high capacitance at low current density, its performance degrades significantly at high current density (Figure 5b). However, with the incorporation of pristine graphene sheets, a tremendous decrease in relaxation time was observed (Figure 6c), which implies that the ion transport and rate constant get much better. Hence, through the preparation of this composite, we wanted to show that by simply incorporating a conductive base (graphene sheets) one can achieve much higher energy density without losing the power density.

Table S4. Comparison of supercapacitance performance of N-RGO-Gs with other reported graphene-based materials.

Material synthesized	Electrolyte	Capacitance (Fg^{-1})	Surface area (m^2g^{-1})	Rate capability	Reference
Carbon nanofiber paper/graphene	6M KOH	**197 (1.25 A g^{-1})**	**463**	86% (from 1A/g to 2A/g)	15
CNTs/graphene	6M KOH	**401 (1 A g^{-1}), 271 (10 A g^{-1})**	**903**	[a] 68% (from 1A/g to 10A/g)	16
Carbon aerogel/graphene	6M KOH	**205 (1 A g^{-1})**	**792**	[a] 81% (from 1A/g to 5A/g)	17
Carbon nanofibers/graphene	6M KOH	**205 (1 A g^{-1})**	**--**	[a] 50% (from 0.1A/g to 2.5A/g)	18
Activated carbon aerogel/graphene	6M KOH	**300 (1 A g^{-1})**	**1158**	[a] 79% (from 1A/g to 5A/g)	19
Silicon-containing porous carbon nanofibers/graphene	6M KOH	**144.8 (1 mA cm^{-2})**	**437**	[a] 80% (from 1A/g to 20A/g)	20
Porous graphene/activated carbon	6M KOH	**210 (1 mV s^{-1})**	**2106**	--	21
Carbon nanofiber/graphene	6M KOH	**183 (1A g^{-1})**	**480**	[a] 83 (from 1A/g to 10A/g)	22
Carbon nanotube/reduced graphene oxide	6M KOH	**244 (50 mV s^{-1})**	**370**	--	23
Porous CNT-networks/graphene balls	6M KOH	**162.3 (10 mV s^{-1})**	**587**	65.3 (from 10 mV/s to 1000 mV/s)	24
N-doped Activated carbon/graphene oxide	6M KOH	**356.2 (1 A g^{-1}),**	**1957.2**	[a] 83% (from 1A/g to 10A/g)	25
N-RGO-GS	6M KOH	**270 (1 A g^{-1})**	**171**	88% (from 1A/g to 10A/g)	Present work

a: values are not reported in the respective manuscript, but calculated using the given data in the manuscript.

References

1. Hansen, C. M. Hansen Solubility Parameters: A User's Handbook. *CRC Press* (2007).
2. Hernandez, Y., Lotya, M., Rickard, D., Bergin, S. D. & Coleman, J. N. Measurement of Multicomponent Solubility Parameters for Graphene Facilitates Solvent Discovery. *Langmuir* **26,** 3208–3213 (2010).
3. Kunadian I., Lipka S. M., Swartz C. R., Qian D. & Andrewsa R. Determination of Carrier Densities of Boron- and Nitrogen-Doped Multiwalled Carbon Nanotubes Using Mott–Schottky Plots. *J. Electrochem. Soc.* **156**, K110–K115 (2009).
4. Huygens I. M. & Strubbe K. Electrochemical Impedance Study of the Germanium/Electrolyte Interface. *J. Electrochem. Soc.* **155,** F49–F54 (2008).
5. Madou M. J. & Cardon F. Impedance Measurements at the N- and P-Type GaP Single Crystal Electrode. *J. Electrochem. Soc.* **124,** 1623–1627 (1977).
6. Cardon F. & Gomes W. P. On The Determination Of The Flat-Band Potential Of A Semiconductor In Contact With A Metal Or An Electrolyte From The Mott-Schottky Plot. *J. Phys. D: Appl. Phys.* **11** L63–L67 (1978).
7. Nicollian E. H. & Goetzberger A. The Si-SiO2 Interface—Electrical Properties as Determined by the Metal-Insulator-Silicon Conductance Technique. *Bell Syst. Tech. J.* **46,** 1055–1133 (1967).
8. De G. R., Gomes W. P., Cardon F. & Vennik J. On the Interpretation of Mott-Schottky Plots Determined at Semiconductor/Electrolyte Systems. *J. Electrochem. Soc.* **122** 711–712 (1975).
9. Dutoit E. C., Cardon F. & Gomes W. P. Electrochemical Properties of the Semiconducting TiO_2 (Rutile) Single Crystal Electrode. *B. Bunsenges. Phys. Chem.* **80,** 475–481 (1976).

10. Stoller M. D. *et al.* Interfacial Capacitance of Single Layer Graphene. *Energy Environ. Sci.* **4,** 4685–4689 (2011).
11. Laflere W. H., Van Meirhaeghe R. L., Cardon F. & Gomes W. P. On The Frequency-Dependence of the Impedance of N- And P-Type Gallium Arsenide Electrodes. *Surface Sci.* **59,** 401–412 (1976).
12. Wang, K. Lia, L. Zhang, T. & Liu, Z. Nitrogen-Doped Graphene For Supercapacitor With Long-Term Electrochemical Stability. *Energy* **70**, 612–617 (2014).
13. Jeong, H. M. Lee, J. W. Shin, W. H. Choi, Y. J. Shin, H. J. Kang, J. K. & Choi, J. W. Nitrogen-Doped Graphene For High-Performance Ultracapacitors And The Importance Of Nitrogen-Doped Sites At Basal Planes. *Nano Lett.* **11**, 2472–2477 (2011).
14. Wen, Z., et al. Crumpled Nitrogen-Doped Graphene Nanosheets with Ultrahigh Pore Volume for High-Performance. *Supercapacitor* **24**, 5610–5616 (2012).
15. Tai, Z., Yan, X., Lang, J., Xue, Q. Enhancement of Capacitance Performance of Flexible Carbon Nanofiber Paper by Adding Graphene Nanosheets. J. *Power Sources* **199**, 373–378 (2012).
16. Yang, Z.-Y., Zhao, Y.-F., Xiao, Q.-Q., Zhang, Y.-X., Jing, L., Yan, Y.-M., Sun, K.-N. Controllable Growth of CNTs on Graphene as High-Performance Electrode Material for Supercapacitors. *ACS Appl. Mater. Interfaces* **6**, 8497–8504, (2014).
17. Lee, Y.J., Park, H.W., Kim, G.-P., Yi, J., Song, I. K., Supercapacitive Electrochemical Performance of Graphene-Containing Carbon Aerogel Prepared Using Polyethyleneimine-Modified Graphene Oxide. *Curr. Appl. Phys.* **13**, 945–949 (2013).
18. Zhou, Z., Wu, X.-F., Graphene-Beaded Carbon Nanofibers For Use In Supercapacitor Electrodes: Synthesis And Electrochemical Characterization. *J. Power Sources* **222**, 410–416 (2013).
19. Lee, Y.J., Kim, G.-P., Bang, Y., Yi, J., Seo, J.G., Song, I.K. Activated Carbon Aerogel Containing Graphene as Electrode Material for Supercapacitor. *Mater. Res. Bull.* **50**, 240–245, (2014).
20. Kim, S.Y., Kim, B.-H., Yang, K.S., Oshida, K. Supercapacitive Properties of Porous Carbon Nanofibers via the Electrospinning of Metal Alkoxide-Graphene in Polyacrylonitrile. *Mater. Lett.* **87**, 157–161 (2012).
21. Zheng, C., Zhou, X., Cao, H., Wang, G., Liu, Z. Synthesis of Porous Graphene/Activated Carbon Composite with High Packing Density and Large Specific Surface Area for Supercapacitor Electrode Material. *J. Power Sources* **258**, 290–296 (2014).
22. Dong, Q., Wang, G., Hu, H., Yang, J., Qian, B., Ling, Z., Qiu, J. Ultrasound-Assisted Preparation Of Electrospun Carbon Nanofiber/Graphene Composite Electrode For Supercapacitors. *J. Power Sources* **243**, 350–353 (2013).
23. Zeng, F., et al. Multilayer Super-Short Carbon Nanotube/Reduced Graphene Oxide Architecture for Enhanced Supercapacitor Properties. *J. Power Sources* **247**, 396–401 (2014).
24. B.S. Mao, Z. Wen, Z. Bo, J. Chang, X. Huang, J. Chen, Hierarchical Nanohybrids with Porous CNT-Networks Decorated Crumpled Graphene Balls for Supercapacitors. *ACS Appl. Mater. Interfaces* **6**, 9881–9889 (2014).
25. Xie Q., Sandwich-Like Nitrogen-Enriched Porous Carbon/Graphene Composites as Electrodes for Aqueous Symmetric Supercapacitors with High Energy Density. *Electrochimica Acta* **189**, 22–31 (2016).
